# Early Response Monitoring Following Radiation Therapy by Using [^18^F]FDG and [^11^C]Acetate PET in Prostate Cancer Xenograft Model with Metabolomics Corroboration

**DOI:** 10.3390/molecules22111946

**Published:** 2017-11-10

**Authors:** Yi-Hsiu Chung, Cheng-Kun Tsai, Chiun-Chieh Wang, Hsi-Mu Chen, Kuan-Ying Lu, Han Chiu, Yu-Chun Lin, Tzu-Chen Yen, Gigin Lin

**Affiliations:** 1 Center for Advanced Molecular Imaging and Translation (CAMIT), Chang Gung Memorial Hospital, Taoyuan 333, Taiwan; stella720905@gmail.com (Y.-H.C.); chiuhan911@gmail.com (H.C.); 2 Department of Nuclear Medicine, Chang Gung Memorial Hospital, Taoyuan 333, Taiwan; klem.tsaick@gmail.com; 3 Department of Radiation Oncology, Chang Gung Memorial Hospital, Taoyuan 333, Taiwan; jjwang@cgmh.org.tw; 4 Imaging Core Lab, Department of Medical Imaging and Intervention, Institute for Radiological Research, Chang Gung Memorial Hospital at Linkou and Chang Gung University, Linkou Medical Center, 5 Fuhsing Street, Guishan, Taoyuan 333, Taiwan; mulder1520@gmail.com (H.-M.C.); fantacy52317@gmail.com (K.-Y.L.); jack805@gmail.com (Y.-C.L.); 5 Clinical Metabolomics Core Lab, Chang Gung Memorial Hospital at Linkou, Taoyuan 333, Taiwan

**Keywords:** [^11^C]Acetate, cancer metabolism, 2-deoxy-2-[^18^F]fluoro-d-glucose, nuclear magnetic resonance, positron emission tomography, total lesion glycolysis, radiation therapy

## Abstract

We aim to characterize the metabolic changes associated with early response to radiation therapy in a prostate cancer mouse model by 2-deoxy-2-[^18^F]fluoro-d-glucose ([^18^F]FDG) and [^11^C]acetate ([^11^C]ACT) positron emission tomography, with nuclear magnetic resonance (NMR) metabolomics corroboration. [^18^F]FDG and [^11^C]ACT PET were performed before and following irradiation (RT, 15Gy) for transgenic adenocarcinoma of mouse prostate xenografts. The underlying metabolomics alterations of tumor tissues were analyzed by using ex vivo NMR. The [^18^F]FDG total lesion glucose (TLG) of the tumor significant increased in the RT group at Days 1 and 3 post-irradiation, compared with the non-RT group (*p* < 0.05). The [^11^C]ACT maximum standard uptake value (SUVmax) in RT (0.83 ± 0.02) and non-RT groups (0.85 ± 0.07) were not significantly different (*p* > 0.05). The ex vivo NMR analysis showed a 1.70-fold increase in glucose and a 1.2-fold increase in acetate in the RT group at Day 3 post-irradiation (*p* < 0.05). Concordantly, the expressions of cytoplasmic acetyl-CoA synthetase in the irradiated tumors was overexpressed at Day 3 post-irradiation (*p* < 0.05). Therefore, TLG of [^18^F]FDG in vivo PET images can map early treatment response following irradiation and be a promising prognostic indicator in a longitudinal preclinical study. The underlying metabolic alterations was not reflected by the [^11^C]ACT PET.

## 1. Introduction

Radiation therapy is widely used in the primary treatment for cancer, either alone or as a part of adjuvant or combination therapy [1,2]. Accurate spatial localization is critical for optimal planning [3], and monitoring early radiation response can improve treatment efficacy and prognostic prediction [4,5,6]. Ionized radiations induce serial cellular and tissue responses, such as reactive oxygen species system [7,8], nuclear DNA damage [9], inflammation [10], apoptosis [11] and necrosis [12,13]. All of the biological alternations potentially serve as imaging biomarkers for spatial localization and monitoring early radiation response [14,15,16]. The Response Evaluation Criteria in Solid Tumor (RECIST) criteria suggests that the time to evaluate treatment response of solid tumors is 6–8 weeks following treatment [17]. The response to radiation therapy is not known until the therapeutic course completes. With the advancement of radiation therapy-high hazard dose accumulating within the target lesion and preserving the surrounding normal tissue [18], there is increasingly important to monitor response at the earliest time point as possible. 

2-deoxy-2-[^18^F]fluoro-d-glucose ([^18^F]FDG) positron emission tomography [19] is a means of quantitative measuring glucose metabolism, as reflected by the cellular uptake and accumulations of [^18^F]FDG in vivo [20], and has been extensively used in oncology for diagnosis and assessment of the treatment response [21,22]. Whether [^18^F]FDG PET can evaluate the efficacy of radiation therapy, particularly in the early treatment period, however, remains controversial [23,24,25]. Maximum standard uptake value (SUVmax) has a significantly improved reproducibility as compared to mean standard uptake value (SUVmean), since the maximum value within a region of interest (ROI) is typically invariant with respect to small spatial shifts of the ROI. Despite the image scan and animal physiological conditions, all factors were consistent during the experiments such as a threshold setting, animal fasting and warming to reduce the variability of SUV measurement. The previous studies reported that the actual variability of SUVmax of [^18^F]FDG PET is greater than 15% to 20% due to individual physiological status and inadequacy imaging setup [26,27]. A novel metabolic volumetric quantitative index, total lesion glucose (TLG) [28] has been reported a more reliable and promising prognostic index [29,30]. An alternative metabolic PET tracer, [^11^C]acetate ([^11^C]ACT), has recently emerged for the diagnosis of prostate cancer [31] and for monitoring the hormone treatment response in tumor-bearing animal models [32]. Acetate is the main building block of acetyl-coenzyme A (acetyl Co-A), which is important in conveying the carbon atoms within acetyl group to the citric acid cycle for energy production. The combination of dual metabolic PET tracers, namely [^18^F]FDG and [^11^C]ACT, provides potential to elucidate a timeline of the complex metabolic alterations in tumors following radiation therapy in vivo study. Furthermore, nuclear magnetic resonance (NMR) metabolomic approach has emerged as an important tool to globally profile tumor metabolites [33], to gain insights into how tumors orchestrate metabolic alterations in response to radiation therapy. Together, the metabolic imaging and metabolite analysis lend further support to the hypothesis that early tumor metabolic response to radiation therapy plays an important role in prognostic prediction, alternative treatment planning, and synergistic antitumor strategies. 

The aim of this study was to characterize the metabolic changes associated with early response to radiation therapy in a prostate cancer mouse model by using dual-tracer [^18^F]FDG and [^11^C]ACT PET, with in vitro cells and ex vivo tissue NMR metabolomics corroboration. 

## 2. Results 

### 2.1. Early Changes on [^18^F]FDG PET of Tramp-C Prostate Tumors Following Irradiation

**Figure 1:**
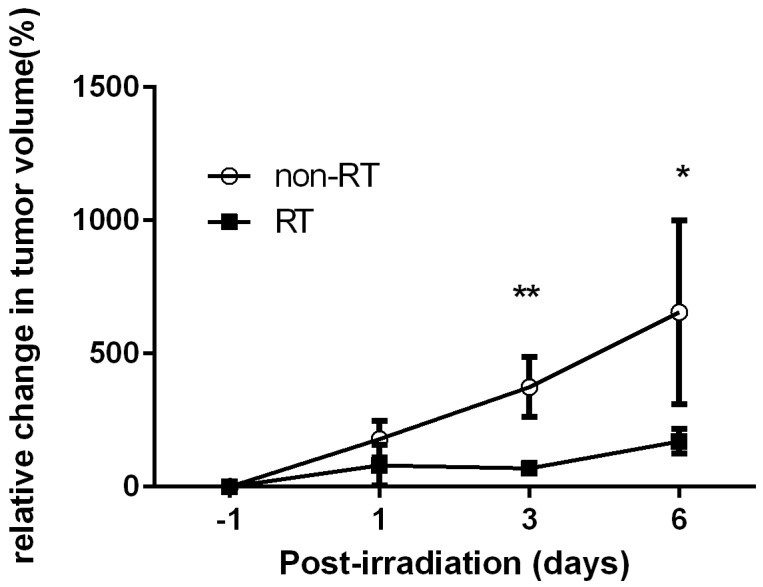
The changes in tumor size of Tramp-C prostate tumor-bearing mice. Mice with Tramp-C tumors were treated with 15Gy of single-fractionated irradiation. The tumors of irradiated mice were significant decreased in Day 3 and Day 6. * *p* < 0.05; ** *p* < 0.01.

**Figure 2:**
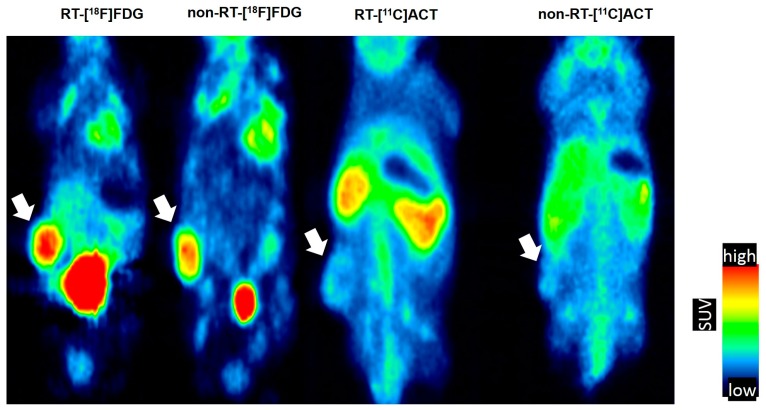
Coronal view of a representative [^18^F]FDG and [^11^C]Acetate ([^11^C]ACT) PET scans in non-RT mouse and Day 3-RT mouse. The relative lower tumor uptake in [^11^C]ACT PET. Tumors are indicated by white arrows.

**Figure 3:**
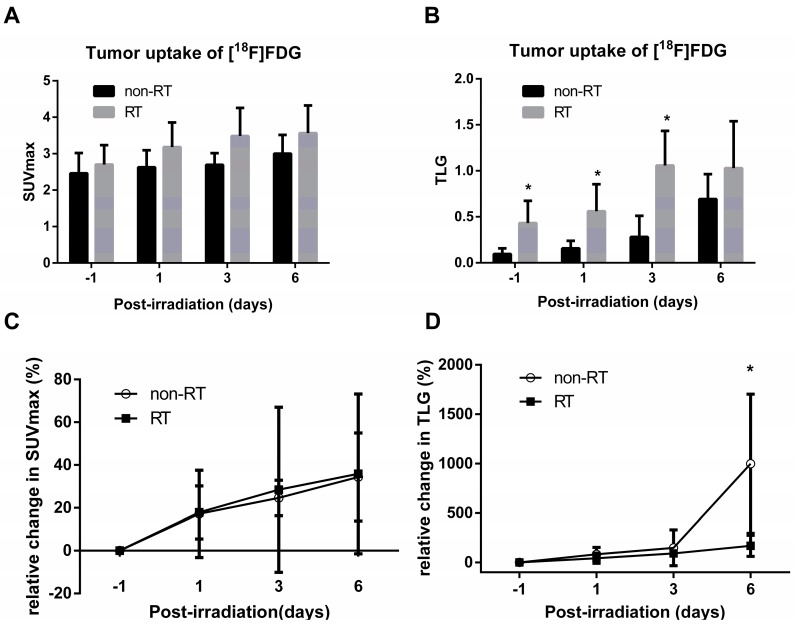
Semi-quantification of [^18^F]FDG. Averaged **A**, SUVmax **B**, TLG of tumors and averaged **C** SUVmax and **D** TLG changes (%) from baseline in RT and non-RT groups. * *p* < 0.05. At post-irradiation Day 1 and Day 3, the SUVmax and TLG of tumors were higher than that of non-RT tumors. On the contrary, the degree of ΔTLG of RT tumor was significantly lower at Day 6.

Significantly reduced size of the tumors were found following irradiation at Days 3 and 6, as compared with non-RT controls *(p* < 0.01 and *p* < 0.05, respectively, Figure 1). The tumors [^18^F]FDG PET images in Day 3 days comparing the RT vs. non-RT tumors are represented (Figure 2). The quantitative results were reported in Figure 3. The [^18^F]FDG TLGs for the RT tumors were 0.56 ± 0.29 (*p* < 0.05), 1.06 ± 0.38 (*p* < 0.05), and 1.03 ± 0.5 (*p* = 0.26) at post-irradiation Day 1, 3 and 6, significantly higher as compared with non-RT tumors, 0.16 ± 0.08, 0.28 ± 0.23 and 0.69 ± 0.27. The [^18^F]FDG SUVmax for RT tumors were higher than non-RT tumors at various time points, albeit not statistically significant. A significant statistical difference in the percentage change in [^18^F]FDG TLG (ΔTLG) was observed between the groups at post-irradiation Day 6 (*p* < 0.05), which corresponding to tumor size measurement by the caliper measurements. The ΔTLG showed a good correlation with the Δ tumor size (*r* = 0.73, *p* < 0.01, Supplementary Materials Figure S1). Details values of volume of interest (VOI) of tumors, SUVmean and TLG of individual tumors were listed (Supplementary Materials Table S1).

### 2.2. No Remarkable Changes on [^11^C]ACT PET of Tramp-C Prostate Tumors Following Irradiation

To examine whether the alterations of glycolysis at Day 3 also involved in the fatty acid metabolism in vivo, [^11^C]ACT PET of Tramp-C prostate tumors following irradiation was carried out. We found no statistically significant differences between RT and non-RT tumors in terms of the [^11^C]ACT SUVmax of (0.83 ± 0.02 vs. 0.85 ± 0.07, *p* = 0.61) and TLA of 0.16 ± 0.06 vs. 0.17 ± 0.07, *p* = 0.9). The time-activity curve of prostate tumor in the dynamic [^11^C]ACT PET within 1 h showed the maximum tumor uptake within 1-h dynamic scan was at the 10 min after tracer injection (Supplementary Materials Figure S2).

### 2.3. In Vitro Cells NMR Corroboration

To understand the metabolic contribution of Tramps-C cancer cells, we conducted in vitro cells NMR metabolomics analysis. Following irradiation treatment, the number of Tramp-C cells was reduced by 12.5% at 6 h and 37.5% at 24 h compared with the non-irradiation group. The analysed aqueous metabolites (glucose, lactate, glutamate and glutamine) and the lipophilic metabolite (CH_2_)*_n_* of irradiation-treated Tramp-C cancer cell increased by an average of 50% at 6 h (*p* < 0.05 for glucose), and these metabolites continued to increase and doubled at 24 h (*p* < 0.05 for all) relative to the non-irradiated cells. Acetate decreased at 6 h and significantly increased at 24 h (*p* < 0.001 in the irradiated cells compared with the non-irradiated cells (Supplementary Materials Table S2). 

### 2.4. Ex Vivo Tissue NMR Corroboration

**Figure 4:**
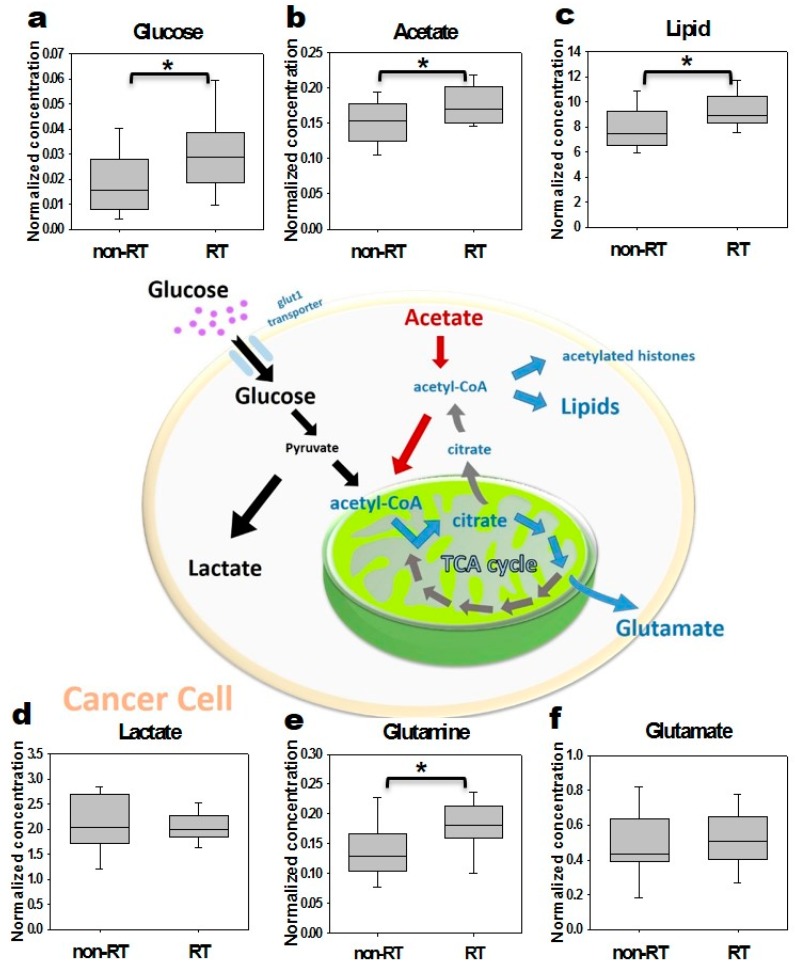
The aqueous and lipophilic metabolites in non-irradiated and irradiated tumors Day 3 post-irradiation in the glycolysis process based on NMR analysis. The significant increased metabolites, glucose, acetate, lipid as well as glutamine were detected in the tumors with radiation in 3 days. * *p* < 0.05.

The levels of aqueous and lipophilic metabolites associated with the glycolysis pathway in irradiated and non-irradiated tumor ^1^H-NMR-based spectrum analyses was shown. In particular, the ratios of the irradiation to the non-irradiation group in the levels of glucose and acetate increased 1.7-fold (*p* < 0.05) and 1.2-fold (*p* < 0.05) at Day 3 (Figure 4), respectively. However, the level of acetate decreased at Day 7 after radiation, and the level of glucose increased 1.9-fold (*p* > 0.05 for both, data not shown).

### 2.5. Histopathology and Western Blotting Verifications

**Figure 5:**
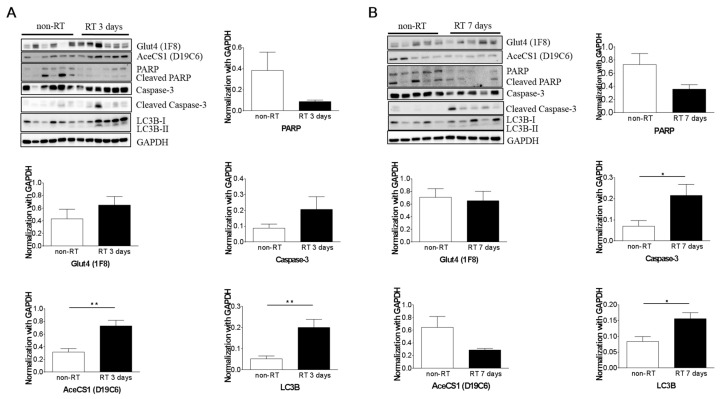
The Glut4, AceCS1, PARP, Caspase-3 and LC3 protein levels at Day 3 (**A**) and Day 7 (**B**) post-irradiation compared with the non-irradiated group. AceCS1 is overexpressed in RT 3 days tumors. No significant difference of Glut4 overexpression was found in the RT-tumors with Day 3 and Day 7 post-irradiation. * *p* < 0.05.

**Figure 6:**
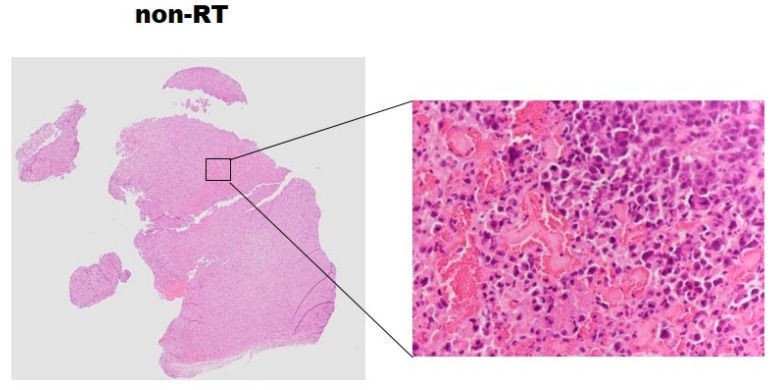

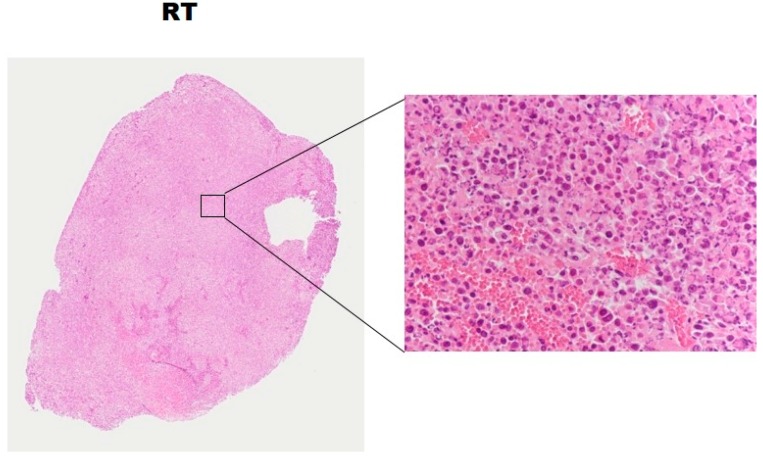
Hematoxylin and eosin stained tumor slices representative of the non-irradiated tumor and irradiated tumor at Day 3 post-irradiation. There is no majority of the necrotic area in the irradiated group.

Cytoplasmic acetyl-CoA synthetase (AceCS1) and Light chain 3 (LC3) were overexpressed at Day 3 post-irradiation compared with the non-irradiation group (*p* < 0.05). Caspase-3 and Light chain 3 were overexpressed at Day 7 post-irradiation compared with the non-irradiation group (Figure 5, *p* < 0.05). Nevertheless, at Day 3 and 7 post-irradiation, there was no significant difference in Glut4 expression between the irradiated and non-irradiated groups, even though a slightly overexpression in irradiated tumors. None of the tumors in this study exhibited central necrosis on the histology analysis (Figure 6).

## 3. Discussion

The present study characterized metabolic alterations in glycolysis and fatty acid metabolism in vivo using [^18^F]FDG and [^11^C]ACT with ex vivo NMR metabolomics analysis in a prostate cancer mouse model following radiation therapy. The TLG of the RT tumors showed a 3.5-fold significant increase as early as Day 1 and persisted to Day 3, compared to the non-RT tumors. The increased uptake of [^18^F]FDG were supported by the elevated levels of glucose by NMR analysis of tumors. Although not detected by [^11^C]ACT PET imaging, the augmented acetate utilization following radiation was detected by NMR analysis and supported by the over-expressed AceCS1. [^18^F]FDG uptake has been shown to decrease in the irradiated tumors [34]. Indeed, the increased [^18^F]FDG uptake at early time point following RT, as observed in SUVmax and TLG, was an intriguing finding. Therefore, we further interrogated tumor metabolism by NMR metabolomics analysis which supported the increased tumor glucose level (Figure 4). The plausible explanation was inflammation following RT as demonstrated on Hematoxylin and eosin stained tumor slices. Another contributing factor was the activation of cellular responses including autophagy, evidence of the significantly increased LC3B-II level following RT on both Day 3 and Day 7. The tumor SUVmax on [^18^F]FDG PET are commonly used for cancer diagnosis and staging in clinical settings [26]. Both [^18^F]Fluorothymidine and [^18^F]FDG have shown to decrease in the irradiated tumors of human head and neck xenograft mice through post-irradiation Day 5 to Day 15 [34]. In another study on primary and metastatic colorectal xenograft mice, significant decreases were observed in [^18^F]Fluorothymidine of tumors at Day 1 after irradiation, better than ^18^F-FDG in monitoring the colorectal tumor response to 24 h radiation treatment [6]. We found that the tumor TLG, taking into account volume-based and glucose metabolism, demonstrates the ability of early prediction for the tumor response to radiation treatment, which was not observed in the SUVmax of tumors. Our results were supported by Dijk et al. [35,36], showing that the SUVmax [^18^F]FDG uptake not significantly changed in murine squamous cell carcinoma at Day 7 after a dose of 10 or 20 Gy irradiation. Compared to SUVmax, TLG was found to be a better prognostic indicator of oncological outcomes when [^18^F]FDG PET/CT performed during radiation therapy in soft-tissue sarcoma and locally advanced head and neck squamous cell carcinoma [29,30].

Although we did not investigate the inflammation timeline after irradiation, our NMR metabolomic analysis demonstrated the metabolic alteration of cancer cells happened as early as 6 h following radiation therapy, demonstrating two-fold increases in glucose and lactate. The levels of glucose, lactate, glutamate, glutamine, acetate and lipophilic metabolites continued to elevate at 24 h in Tramp-C cancer cells. Significant increases in glucose, glutamine, acetate, and lipids were noted in the tumor at Day 3 following radiation therapy. Our data and the literature evidence demonstrated that the underlying metabolic adaptations to radiation therapy open a window for translatable imaging biomarkers for timely assessment of the response to radiation [37]. [^11^C]ACT have been employed in the detection of primary or recurrent prostate cancer with a 59–79% of sensitivity and 72–98% of specificity [38]. [^11^C] ACT PET images have performed in tumor detection for tumors that are most-liked the fatty acid synthesis pathway [38]. In the present study, however, the [^11^C]ACT uptake was limited in xenograft prostate tumors, despite the dynamic scans and optimal time point selection, the level of [^11^C]ACT was still beyond the detection limits of PET. Nonetheless, the cytoplasmic acetyl-CoA synthetase of the tumor, synthesizing acetyl-CoA used for fatty acid and lipid biosynthesis, was significant increasing at Day 3 post-irradiation. Further isotope labeled study will be conducted to elucidate the alterative origins of the elevated acetate in tumors from glucose or glutamine.

The drawback of the study is the tumor size control in the pre-irradiation. We did not group the irradiated and non-irradiated mice carefully. The tumor sizes of the irradiated mice bearing are larger, which might impact the maximum SUV for tumors at the beginning time point as well as TLG measurement as the TLG is related to tumor size and mean SUV. As a result, we cannot rule out that the significant difference of TLG between the groups in post-irradiation Day 1 and 3 is not from tumor size influence. The difference between the TLG of RT versus non-RT groups in Figure 3B was partly related to the larger tumor volumes of RT group than non-RT group at the initial time point. Therefore, we used the relative TLG changed to compared with each other in Figure 3D. Despite the discrepancy in tumor size in the irradiation and the non-irradiation groups, the NMR metabolomic analysis demonstrates the increased glucose and fatty acid synthesis in the early tumors response to radiation therapy. 

A potential translation from this study is the clinical application of advanced radiation therapy, such as proton therapy. The high hazard dose is accumulated within the tumor, with less than 60% of the radiation dose distributed in the surrounding tissue. This leads to better therapeutic efficacy and fewer side effects [18]. Molecular imaging monitors the early response to proton therapy based on the biological features associated with metabolism and microenvironment alternation, allowing better patient management and timely adaptive treatment planning. In addition, the short-lived positron emitters produced during proton irradiation provide an attractive option for PET imaging to verify in vivo dose delivery [39]. Our findings in metabolite changes using NMR analysis are encouraging for the development of molecular tracers to detect metabolite changes, allowing us to quantitatively determine changes in metabolites for reflecting early tumor radiation response.

## 4. Materials and Methods

### 4.1. Cell Culture

Transgenic adenocarcinoma of mouse prostate (Tramp-C) cancer cells (CR UK Cell Services, South Mimms, Herts, UK) served as a tumor model in this study. Cells grew in Dulbecco’s Modified Eagle Medium (DMEM, high glucose, Gibco, NY, USA), 10% fetal bovine serum (FBS, Gibco) and 1% antibiotics (penicillin/streptomycin, Gibco) growth media. Cells were treated with 3 Gy irradiation 24 h after the cells were seeded. The irradiated cells were incubated for 6 and 24 h. Non-irradiated cells plated served as a control group. Then, the cells were neutralized with a medium and centrifuged for 5 min at 1100 rpm. The medium was discarded, and the pellet was suspended using the known volume of phosphate-buffered saline (pH 7.4). The suspension procedure described above was repeated twice.

### 4.2. Animal Experiment

C57Bl/6 mice (approximately 6–8 weeks old with a body weight of 20–25 g) were obtained from the National Laboratory Animal Center (Taipei, Taiwan). All animal experiments were performed according to a protocol approved by the Institutional Animal Care and Use Committee, Chang Gung University and Chang Gung Memorial Hospital, Taiwan (IACUC 2012092401). The prostate cancer cells (3 × 10^6^) were subcutaneously inoculated into the flank region of male mice. Tumor growth was monitored by caliper measurements. When the tumors reached long-axis diameters of 6–10 mm, the [^18^F]FDG (*n* = 11) and [^11^C]ACT (*n* = 5) PET was conducted before 15 Gy irradiation. Irradiated (RT) and non-RT tumor-bearing mice were scanned with [^18^F]FDG PET at Days 1 (RT, *n* = 6 vs. non-RT, *n* = 5), 3 (RT, *n* = 4 vs. non-RT, *n* = 3) and 6 (RT, *n* = 6 vs. non-RT, *n* = 5) post-irradiation. Dynamic [^11^C]ACT PET images were obtained at Days 3 post-irradiation (*n* = 4) in other tumor-bearing mice. The NMR metabolic analyses were performed at Days 3 and 7 following radiation therapy in the ex vivo study. Unless otherwise specified, all results were from the RT treatment vs. non-RT control comparisons (*n* ≥ 4 in each group). 

### 4.3. Irradiation Setup

The irradiation experiment was performed as previously described [40]. Briefly, the Tramp-C cells in the Petri dish were placed under the collimated beam, and 3 Gy of single-fractionated irradiation was performed. In irradiation setup of animal procedure, mice were anesthetized with a mixture of ketamine and xylazine during irradiation, and 15 Gy of irradiation was performed with a 0.5-cm bolus on the surface. Irradiation was conducted using the 6 MV Novalis system (BrainLab, Feldkirchen, Germany) with a 2-cm stereotactic radiosurgery cone. 

### 4.4. [^18^F]FDG and [^11^C]ACT PET and Imaging Analysis

The tumor-bearing mice were imaged using an InveonTM system (Siemens Medical Solutions Inc., Malvern, PA, USA) at Chang Gung Memorial Hospital, Taiwan. The mice underwent a 10-min image acquisition in the prone position in 60 min after receiving 6.75–8.1 MBq of [^18^F]FDG via intraorbital injection. Frequent intravenous injecting of [^18^F]FDG for serial monitoring response following radiation therapy may cause the damage, inflammation or burse of the tail vein. Hence, we used intraorbital injection to reduce the variation from the damaged tail vein [19,41]. [^11^C]ACT dynamic images of the mice were acquired for 1 h (12 frames × 5 min) using 22.2 MBq. The dynamic [^11^C]ACT PET images were summed as an average image (10–25 min, maximum tumor uptake within 1-h scan) for image analysis. The mice were anesthetized with 2% isoflurane and placed near the center of the field of view. The infrared heat lamp was used during scanning to prevent the mice from hypothermia. Reconstructed images matrix size is 128 pixels × 128 pixels × 159 slices with 0.39 mm × 0.39 mm × 0.80 mm using 2D ordered-subset expectation maximum iterative method.

To compare with clinical quantitative PET image standard, the radioactivity concentration of microPET images was converted to standard uptake values (SUV) by multiplying the individual body weight, and the dividing injected dose. The [^18^F]FDG uptake and [^11^C]ACT of the tumor was expressed as the maximum standard uptake value (SUVmax). To measure the mean SUV and volume of the whole tumor, regions of interests (ROIs) were determined semi-automatically with a threshold of (maximum–minimum) of contoured ROIs × 50% [42]. Readers were blinded for RT and non-RT groups and we used the consistent method to analyze the tumor uptake in two groups. The total lesion glycolysis (TLG) is defined as SUVmean of [^18^F]FDG x ROI selected tumor volume. The total lesion acetate (TLA) is defined as SUVmean of [^11^C]ACT ROI selected tumor volume. Use multi-contours ROI to reduce the misselected the value less than the threshold in the center of the tumors. The SUVmax and TLG in sequence scans were used to defined ΔSUVmax and ΔTLG in percentage as follows:

$$\Delta \mathrm{SUVmax}=\left[\frac{{\mathrm{SUV}}_{\mathrm{Day}-1}-{\mathrm{SUV}}_{\mathrm{time}\;\mathrm{points}}}{{\mathrm{SUV}}_{\mathrm{Day}-1}}\right]\times 100\%$$

$$\Delta \mathrm{TLG}=\left[\frac{{\mathrm{TLG}}_{\mathrm{Day}-1}-{\mathrm{TLG}}_{\mathrm{time}\;\mathrm{points}}}{{\mathrm{TLG}}_{\mathrm{Day}-1}}\right]\times 100\%$$

All image analyses were conducted by using PMOD version 3.2 (PMOD Technologies Ltd., Zurich, Switzerland).

### 4.5. NMR Metabolomic Analysis 

Immediately following three initial 0.9% saline washes at 4 °C, cells covered with ice-cold methanol were scraped from each plate and placed on ice, and ice-cold water and chloroform were added in succession with vortexing between additions. In the ex vivo study, when tumors grew to approximately 10 mm in diameter, they were subjected to irradiation. At Day 3 (irradiation, *n* = 15; non-irradiation, *n* = 15) and Day 7 (irradiation, *n* = 9; non-irradiation, *n* = 8) post-irradiation, the tumors were removed to carry out the dual-phase extraction and NMR study as described following. NMR spectra were acquired using a Bruker Avance II HD 600 MHz spectrometer (Bruker Biospin GmbH, Rheinstetten, Germany) operating at 600.13 MHz and equipped with a TXI CryoProbe (Bruker Biospin GmbH, Rheinstetten, Germany) at 310 K. Two types of ^1^H-NMR spectra were acquired: NOESY and Carr-Purcell-Meiboom-Gill (CPMG) pulse sequence. The analyzed aqueous metabolites were glucose, lactate, acetate, glutamine, and glutamate; the analyzed lipophilic metabolite was a long-chain lipid component (CH_2_)*_n_*. NMR spectral analyses were performed with Bruker TopSpin software (Version 3.2, Bruker Biospin GmbH, Rheinstetten, Germany), and the integrated area of the metabolites was calculated with the spectra of radiation- and non-radiation-treated Tramp-C cells. The aqueous and lipophilic metabolic concentrations were normalized with the cell numbers in each cell culture dish. The ratio of the metabolic concentrations of radiation- and non-radiation-treated Tramp-C cells was determined. The metabolic concentrations of the NMR spectra were computed by the internal reference of sodium 3-trimethylsilyl-2,2,3,3-tetradeuteropropionate (TSP) in the aqueous phase and of tetramethylsilane in the lipophilic phase. The metabolic concentration extracted from the tumor was normalized with the weight of the tumor tissue. 

### 4.6. Western Blotting and Histologic Assay

Tumor lysates were analyzed by western blotting, as described previously [43]. Tumor lysate protein was transferred onto Immobilon-P membranes (Millipore; Bedford, MA, USA). Blots were incubated with Glut4 (1F8) Mouse mAb, AceCS1(D19C6) Rabbit mAb, caspase-3 antibody, cleaved-PARP, or LC3B antibody. GAPDH was used as a loading control. (All above antibodies from Cell Signaling, Beverly, MA, USA). The membranes were then incubated with the anti-rabbit secondary antibody (GE Healthcare, Little Chalfont, UK). Specific-binding antibody–target protein interactions were detected using enhanced chemiluminescence plus reagents (Amersham Biosciences, Chalfont St Giles, UK) and exposure to either Hyperfilm ECL (Amersham) or X-OMAT Kodak (Kodak, Rochester, NY, USA) autoradiography film. Tumors with 3 days post-irradiation in the irradiated and non-irradiated groups were removed, frozen and embedded, sliced and stained with hematoxylin and eosin stain [43]. In a separate group of mice, 3 tumors of each group were included.

### 4.7. Statistical Analysis

Statistical analyses were performed using Prism software (Version 6, GraphPad, La Jolla, CA, USA). The statistical significance of irradiation-induced alternative metabolites in cancer cells and extracted tumors; the SUVmax, TLG and TLA changes in the whole tumor of the irradiation group were compared with the non-irradiation group using Mann-Whitney test. Correlations were tested using the Pearson correlation coefficient. A *p*-value of 0.05 or less was considered statistically significant.

## 5. Conclusions

In conclusion, TLG of [^18^F]FDG in vivo PET images can map early treatment response following irradiation and be a better prognostic indicator in a longitudinal preclinical study. [^11^C]ACT is not suitable for evaluation of Tramp-C tumor acetate response to radiation therapy in vivo. With concordant metabolic changes detected by NMR technique in vitro and ex vivo studies, the early tumor response to radiation therapy could be characterized more completely.

## Supplementary Materials

The following are available online. Figure S1: The positive correlation between ΔTLG and Δtumor size is shown (*r* = 0.73, *p* < 0.01), sample deriving from the RT group in days 1, 3 and 6. Table S1: Tables of volumes of VOIs, SUVmean of [^18^F]FDG, TLG and ΔTLG in RT-tumors and non-RT tumor at the varying time points. Figure S2: A time-activity curves of [^11^C]Acetate in muscle, kidney, liver and tumor in a mouse in one hour. Table S2: Summarize the in vitro cells and ex vivo tumor tissue NMR data.
