# Supplementary material for: Early Response Monitoring Following Radiation Therapy by Using [18F]FDG and [11C]Acetate PET in Prostate Cancer Xenograft Model with Metabolomics Corroboration
Source: Molecules. 2017 Nov 10;22(11):1946. doi: 10.3390/molecules22111946 (PMC6150287; doi:10.3390/molecules22111946)
Supplement: Supplementary file 1 [file molecules-22-01946-s001.pdf]

Supplemental data

Figure S1

The positive correlation between ΔTLG and Δtumor size is shown in the below figure with r=0.73 (p <0.05), sample deriving from the RT group in days 1,3, and 6.

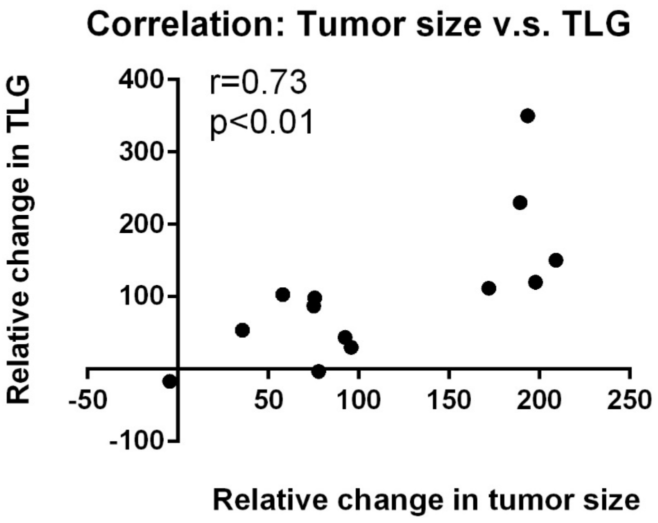

Table S1

Tables of volumes of VOIs, SUVmean of [^{18}F]FDG, TLG and ΔTLG in RT-tumors and non-RT tumor at the varying time points.

RT tumor #	Time point (Day Post-RT)	ROI tumor volume(ccm)	SUVmean	TLG	ΔTLG(%)
Tumor1	-1	0.38	1.67	0.63	0
	1	0.43	2.25	0.97	53.97
	3	0.51	2.30	1.18	87.30
	6	0.55	2.89	1.58	150.79
Tumor2	-1	0.20	1.69	0.34	0
	1	0.19	1.73	0.33	-2.94
	3	0.30	2.32	0.69	102.94
	6	0.18	2.72	0.49	44.12
Tumor3	-1	0.40	1.94	0.77	0
	1	0.32	1.96	0.64	-16.88
	3	0.53	2.91	1.53	98.70
	6	0.62	2.63	1.63	111.69
Tumor4	-1	0.08	1.36	0.1	0
	1	0.14	1.66	0.22	120
	3	N/A	N/A	N/A	N/A
	6	0.23	1.91	0.45	350
Tumor5	-1	0.15	1.95	0.3	0
	1	0.18	2.13	0.39	30
	3	N/A	N/A	N/A	N/A
	6	0.36	2.09	0.99	230
Tumor6	-1	0.19	2.40	0.46	0
	1	0.28	2.92	0.81	76.09
	3	0.46	1.80	0.83	80.43
	6	0.56	1.84	1.04	126.09

Non-RT tumor #	Time point (corresponding Day)	ROI tumor volume(ccm)	SUVmean	TLG	ΔTLG(%)
Tumor7	-1	0.05	1.53	0.07	0
	1	0.07	1.30	0.09	28.57
	3	0.14	2.14	0.3	328.57
	6	0.31	2.08	0.64	814.29
Tumor8	-1	0.04	1.46	0.06	0
	1	0.08	1.74	0.14	133.33
	3	0.03	1.62	0.04	-33.33
	6	0.25	1.68	0.42	600
Tumor9	-1	0.06	1.77	0.1	0
	1	0.06	2.01	0.12	20
	3	N/A	N/A	N/A	N/A
	6	0.30	2.10	0.64	540
Tumor10	-1	0.03	1.49	0.05	0
	1	0.07	2.07	0.14	180
	3	N/A	N/A	N/A	N/A
	6	0.43	2.50	1.07	2040
Tumor11	-1	0.09	2.36	0.2	0
	1	0.16	1.90	0.3	50
	3	0.28	1.82	0.5	150
	6	N/A	N/A	N/A	N/A

Footnote:

1. All measurements are rounded off to the 3rd decimal place
2. N/A, not available. The image studies were not performed at the time points.

Figure S2

A time-activity curves of [^{11}C]Acetate in muscle, kidney, liver and tumor in a mouse in one hour

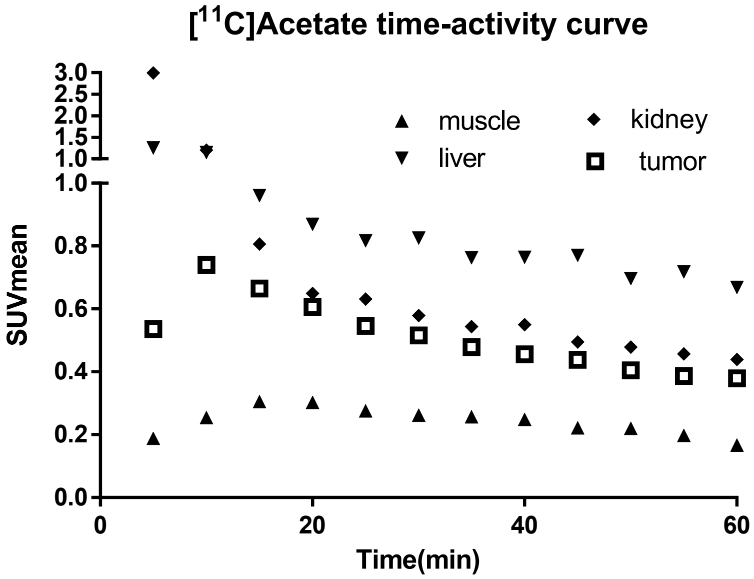

The dynamic [^{11}C]Acetate radioactivity uptake of muscle, kidney, liver and tumor in a mouse in one hour is shown in the above figure. The maximum [^{11}C]Acetate tumor uptake (SUVmean) was in 10-25 min after injection.

Table S2

Summarize the in vitro cells and ex vivo tumor tissue NMR data

metabolites	Cell 6 hr	P-value	Cells 24 hr	*p*-value
**Leucine**	1.60±0.18*	0.011322	2.31±0.14*	0.000043
**Isoleucine**	1.66±0.21*	0.013376	2.46±0.12*	0.000010
**Valine**	1.64±0.20*	0.012272	2.33±0.13*	0.000029
**Lactate**	1.80±0.20*	0.004998	2.97±0.27*	0.000219
**Alanine**	1.52±0.20*	0.047333	1.64±0.10*	0.003770
**Acetate**	0.94±0.09	0.579466	1.96±0.09*	0.000008
**Glutamate**	1.31±0.15	0.101240	1.82±0.08*	0.000033
**Succinate**	1.67±0.24*	0.028050	2.91±0.14*	0.000004
**Glutamine**	1.41±0.16*	0.043810	1.90±0.11*	0.000096
**Glutathione**	1.44±0.26	0.166067	1.60±0.12*	0.003786
**Aspartate**	1.37±0.20	0.136041	1.83±0.09*	0.000085
**Choline**	1.88±0.23*	0.007186	3.65±0.14*	0.000001
**PC**	1.20±0.17	0.341428	1.50±0.08*	0.000957
**GPC**	1.49±0.21	0.065867	2.50±0.09*	0.000001
**Scyllo-Inositol**	1.74±0.59	0.295053	2.51±0.70	0.085073
**Glycine**	1.25±0.19	0.336687	1.94±0.14*	0.001342
**Creatine**	1.28±0.17	0.186497	1.65±0.15*	0.007981
**Phosphocreatine**	1.49±0.32	0.230912	1.39±0.22	0.182515
**Myo-Inositol**	1.47±0.17*	0.036469	1.80±0.10*	0.000437
**Glucose**	1.95±0.32*	0.022616	2.79±0.79*	0.037957
**Fumarate**	1.12±0.29	0.724616	1.10±0.13	0.584518
**Histidine**	1.64±0.22*	0.018618	2.25±0.15*	0.000927
**Tyrosine**	1.58±0.18*	0.013739	2.18±0.11*	0.000029
**Phenylalanine**	1.64±0.20*	0.012418	2.26±0.11*	0.000036
**ADP/ATP**	1.16±0.16	0.428560	1.23±0.08	0.107726
**NADH**	1.27±0.18	0.215854	1.41±0.05*	0.003662
**Formate**	0.89±0.06	0.199555	1.80±0.05*	0.000005

Footnote: *, p-value < 0.05, statistical significant difference between the irradiated cells and non-irradiated cells.
